# Editorial: EGFR-targeted therapy in lung cancer: unraveling molecular regulation, overcoming resistance, and advancing precision clinical practice

**DOI:** 10.3389/fmolb.2026.1936450

**Published:** 2026-08-10

**Authors:** Hongrui Li, Yu-Zhen Zheng, Si-Min Liu, Sen-Yuan Li, Ke-Jun Liu

**Affiliations:** 1 Lineberger Comprehensive Cancer Center, School of Medicine, University of North Carolina at Chapel Hill, Chapel Hill, NC, United States; 2 Department of Thoracic Surgery, ZhuJiang Hospital of Southern Medical University, Guangzhou, China; 3 The Second School of Clinical Medicine, Guangdong Medical University, Dongguan, China; 4 Department of Oncology, The Tenth Affiliated Hospital, Southern Medical University (Dongguan People’s Hospital), Dongguan, China

**Keywords:** EGFR, lung cancer, precision medicine, resistance, targeted therapy

Lung cancer remains the leading cause of cancer-related mortality worldwide, with non-small cell lung cancer (NSCLC) accounting for approximately 85% of all cases. Epidermal growth factor receptor (*EGFR*) mutations are among the most common oncogenic driver alterations in NSCLC and have led to the development of EGFR-targeted tyrosine kinase inhibitors (TKIs), which have become the standard first-line treatment for patients with *EGFR*-mutant NSCLC. Although *EGFR*-TKIs can effectively suppress tumor growth and significantly improve patient outcomes, acquired resistance inevitably develops, resulting in disease progression through diverse molecular mechanisms, including secondary *EGFR* alterations and co-occurring mutations such as *TP53*. Therefore, a better understanding of resistance mechanisms and the development of more effective therapeutic strategies remain urgent clinical priorities. The aim of this Research Topic was to highlight recent advances in overcoming *EGFR*-TKI resistance, including novel therapeutic strategies, emerging resistance mechanisms, predictive biomarkers, and personalized treatment approaches, while providing an overview of current progress and future directions in the field.

*EGFR*-TKIs remain the standard first-line treatment for patients with *EGFR*-mutant NSCLC. However, despite their remarkable clinical efficacy, most patients eventually develop disease progression or metastasis due to acquired resistance. Therefore, identifying effective treatment strategies after TKI resistance remains a major clinical challenge.

Zhang et al. evaluated seven treatment regimens for patients with *EGFR*-mutated advanced NSCLC following TKI progression. The authors found that amivantamab plus lazertinib combined with chemotherapy achieved the longest progression-free survival (PFS) among the seven treatment regimens evaluated, including chemotherapy alone and amivantamab plus chemotherapy. These findings provide valuable evidence for optimizing treatment strategies in patients who develop resistance to first- or second-generation *EGFR*-TKIs.

Nie et al. reported that patients harboring only *EGFR* mutations achieved a longer median PFS than those with concurrent *TP53* or *KRAS* mutations during first-line *EGFR*-TKI treatment. However, following TKI resistance, platinum-based doublet chemotherapy combined with immunotherapy demonstrated greater efficacy in patients with *TP53* or *KRAS* co-mutations, which may be attributable to enhanced tumor immunogenicity and increased sensitivity to immunotherapy.

Zhang et al. investigated the efficacy of immune checkpoint inhibitors (ICIs) in patients with *EGFR* L858R-mutated NSCLC after TKI failure. The analysis showed that combination ICI-based regimens achieved a higher objective response rate (ORR) and prolonged progression-free survival (PFS), although no significant improvement in overall survival (OS) was observed. These findings suggest that selected patients with *EGFR* L858R mutations may benefit from combination immunotherapy following TKI resistance.

Ma et al. evaluated the efficacy and safety of antibody-drug conjugates (ADCs) in patients with *EGFR*-mutant NSCLC after TKI resistance by pooling data from multiple studies. Their analysis demonstrated that ADCs exhibited promising antitumor activity and may represent a potential therapeutic option for overcoming TKI resistance.

Liu et al. compared the effects of early versus delayed radiotherapy in patients with *EGFR*-mutant NSCLC and brain metastases receiving *EGFR*-TKIs. The study demonstrated that radiotherapy administered before *EGFR*-TKI treatment was associated with improved intracranial disease control. In addition, patients harboring *EGFR* exon 19 deletions experienced more favorable outcomes than those with *EGFR* exon 21 L858R mutations.

Zhang et al. described the use of serial patient-derived tumor organoid drug sensitivity testing to guide treatment selection after the failure of first-line *EGFR*-TKI therapy and second-line immunotherapy. The organoid-guided therapeutic strategy resulted in a partial response (PR), highlighting the potential of patient-derived organoids as a promising precision medicine approach for individualized treatment.

Collectively, these studies demonstrate that multiple therapeutic strategies, including combination systemic therapies, radiotherapy optimization, and organoid-guided personalized treatment, offer promising opportunities to improve clinical outcomes after EGFR-TKI resistance.

Acquired resistance remains the central challenge. Eide et al. explored changes in the expression of several immune markers in patients with *EGFR* mutations after first- and second-line *EGFR*-TKI treatment. The expression of these markers varied across EGFR mutation subtypes, which may explain differences in the benefits that patients with lung cancer derive from immunotherapy. However, these findings require validation in larger patient cohorts. Wang et al. reported on a patient with metastatic NSCLC harboring an *EGFR* exon 19 deletion who received an *EGFR*-TKI as first-line treatment. After several lines of TKI treatment, the mutations persisted despite treatment responses. A novel alteration, an *FGFR3-TACC3* fusion, was detected throughout the treatment course. This alteration might have contributed to TKI treatment failure.

*EGFR*-TKIs are among the most commonly used therapies, but their efficacy remains difficult to predict. Patients may or may not benefit from TKI treatment. Certain biomarkers or clinical factors may correlate with disease progression or overall survival. Shalata et al. retrospectively analyzed variant allele frequency (VAF) in patients with *EGFR*-mutated NSCLC. A higher VAF, using a cutoff of 30%, predicted longer overall survival and PFS during first-line osimertinib treatment. Guo et al. found that, among patients with advanced lung adenocarcinoma, a low body mass index (<22.1) and/or low fasting insulin levels were associated with a poor prognosis and shorter overall survival. This study identified new clinical risk factors that may influence the outcomes of TKI treatment for lung cancer. Kim et al. evaluated the efficacy of mobocertinib in patients with NSCLC harboring *EGFR* exon 20 insertion mutations. This *EGFR* exon 20 inhibitor was associated with a modest improvement in median progression-free survival. In addition, baseline ctDNA findings correlated with favorable outcomes and may serve as a prognostic marker.

This Research Topic also highlighted several novel clinical treatment approaches. Cui et al. described a patient with metastatic lung adenocarcinoma. During treatment with amivantamab plus chemotherapy, the patient developed fever and elevated inflammatory markers but also experienced tumor shrinkage. With adequate supportive care, cytokine release syndrome may be associated with tumor control. Luo et al. focused on a patient with *SMARCA4*-deficient NSCLC, an aggressive molecular subtype associated with a poor prognosis. The authors used a series of treatment strategies that alleviated superior vena cava syndrome. Treatment with the third-generation *EGFR*-TKI aumolertinib provided the patient with a period of progression-free survival. Multiple lines of treatment resulted in an overall survival of 2 years. Liu et al. treated a patient with small-cell lung cancer transformed from advanced lung adenocarcinoma with two cycles of olaparib, temozolomide, and atezolizumab, resulting in a partial tumor response. Jin et al. evaluated high-dose furmonertinib in a heavily pretreated patient with advanced *EGFR* L858R-mutant lung adenocarcinoma and metastases to the liver, bones, and brain. The treatment provided sustained clinical benefit, with recovery of consciousness and improvement in daily functioning.

The articles in this Research Topic addressed the challenges of TKI resistance in lung cancer, potential resistance mechanisms, predictive or prognostic factors, and innovative clinical treatment strategies.

Future precision medicine must focus on optimizing targeted combinations, exploring next-generation antibody-drug conjugates alongside immunotherapy regimens, and defining molecular residual disease. Interdisciplinary collaboration remains essential to translate these preclinical insights into durable, survival-extending clinical practices for patients with advanced lung cancer.

